# Time to death and its determinant factors of visceral leishmaniasis with HIV co-infected patients during treatment period admitted at Metema hospital, Metema, Ethiopia: a hospital-based cross-sectional study design

**DOI:** 10.1186/s40794-023-00203-y

**Published:** 2023-10-29

**Authors:** Chekol Alemu, Habitamu Wudu, Getu Dessie, Chalachew Gashu

**Affiliations:** 1 Department of Statistics, College of Natural and Computational Sciences, Gambella University, Gambella, Ethiopia; 2 Department of Statistics, College of Natural and Computational Sciences, Dambi Dollo University, Dembi Dolo, Ethiopia; 3 Department of Statistics, College of Natural and Computational Sciences, Oda Bultum University, Chiro, Ethiopia

**Keywords:** Visceral leishmaniasis, Visceral leishmania with HIV co-infection, Survival analysis, Cox proportional hazard model

## Abstract

**Background:**

Visceral leishmaniasis is caused by the parasites Leishmania donovani spices complex that can spread to internal organs and the disease is fatal with a fatality rate of nearly 100% if left untreated. Visceral Leishmania-HIV (HIV1) coinfection disease is a new clinical form of leishmaniasis very serious disease in the endemic part of the world. It also served as the primary cause of death in the lowlands of Ethiopia with the endemic Humara and Metema that are located near the Sudanese border.

**Methods:**

A total of 153 visceral leishmaniases with HIV co-infection secondary data was taken from the medical chart of patients from January 2015 to January 2021 and a hospital-based cross-sectional study design was carried out to retrieve relevant information. The data entered by SPSS and analysed using STATA version 14 and R4.2.1 statistical software packages using a non-parametric Model, semi-parametric Cox proportional hazard survival models at 5% significance level.

**Result:**

Among the total visceral leishmaniasis with HIV co-infected patients 3.27% were females and 96.73% were males, 19 (12.42%) patients died and 134(87.58%) patients were censored. The Cox proportional hazard model result indicates that severe acute malnutrition, baseline CD4+ cell count ≥100, and underweight significantly contributed to the survival time of a patient. Cox proportional hazard model shows that severe acute malnutrition (HR=4.40027, 95% CI= 2.455061 262.7934, *P*-value=0.007), baseline CD4+cell count ≥100 (HR=0.2714623, 95% CI= 0.0764089 0.9644395, *P*-value=0.044), and Underweight (HR=4.678169, 95% CI= 1.970097 11.10872, *P*-value=0.040) significantly contributed to a shorter survival time.

**Conclusion:**

Visceral leishmaniases with HIV co-infected patients show a large number of deaths occurred in the earlier days of treatment this implies that Visceral leishmaniasis accelerates HIV replication and disease progression death. The researcher suggests that people be aware of the burden posed by those risk factors and knowledgeable about the diseases. So, the researcher recommended that to health workers implement primary health care in those patients and careful consideration of a neglected parasitic disease.

## Background

A neglected parasitic disease known as leishmaniasis is spread by vectors and is brought on by Leishmania protozoa [1]. According to the World Tropical Diseases Research Center, it is classified as one of the top three parasitic diseases along with African trypanosomiasis and dengue fever [2]. Leishmania infections are currently endemic in large areas of the tropics, subtropics and Mediterranean basins spread in more than 98 countries. There are about 350 million people at risk and approximately 12 million people are currently infected. They are also about 2 to 2.5 million new cases reported annually, of which an estimated 600,000 to 1.5 million new cases are cutaneous leishmaniasis (CL). Among all newly reported Leishmania cases, visceral leishmaniasis (VL) is caused by the parasites L. donovani spices with the potential to spread inside the body’s organs and result in death, which contributes 50,000 to 90,000 cases with approximately 20,000 to 50,000 death outcomes worldwide [3–6]. The disease is the most fatal form of Leishmaniasis with a fatality rate of nearly 100%, which occurs within two years if left untreated [7].

East Africa is the second-largest VL (kala-azar) focus, contributing 15% of the estimated annual global burden comprising 0. 2 − 0. 4 million cases [3]. The main populations that contribute to L. donovani are especially heterogeneous, with one population consisting of strains from northern Ethiopia and Sudan and the other population consisting of strains from southern Ethiopia and Kenya [8], and correspond to the regions populated by two different most important sand fly vectors. Phlebotomus Orientalis is the main vector in northern Ethiopia and Sudan while Phlebotomus martini in the South, although other vectors have been also implicated [9]. Phlebotomus celiae Minter and Phlebotomus martini Parrot are to the south of Ethiopia.

In Ethiopia, over 3.2 million people live at risk of infection with up to 4000–7000 new cases of kala-azar per year [3, 10], though the known endemic foci are the Segen- Woito valleys, lower Omo river plains, Lake Abaya area in the southwest, the most important VL endemic areas in Ethiopia are found in the northwest (Metema Humera low land) 1500 m below sea level comprising approximately 60% of the cases [11], and the highest number of VL cases has been previously reported in northwestern Ethiopia including West Armachiho District [10, 12]. In recent years VL has spread to the highlands of the Libo-Kemkem district (south of Gondar) claiming the lives of hundreds of patients [13]. The main clinical features of the visceral leishmaniasis patients are frequent episodes of fever, significant weight loss, splenic and liver enlargement, and anemia are all symptoms of visceral leishmaniasis (which may be serious). In endemic countries, the rK-39 strip test is used to diagnose patients with visceral Leishmania using blood or serum samples [14]. The prevalence of patients with both HIV and VL infection (“HIV-VL coinfection”) in Europe has fallen sharply since 1996 when antiretroviral treatment (ART) became standard. In India and particularly in Africa, HIV-VL coinfection is emerging. The AIDS pandemic has expanded to rural areas where VL is endemic, with cases of HIV-VL coinfection reported in 35 countries [15, 16], among which Ethiopia carries the greatest burden. The affected people are mostly very poor male seasonal migrant workers that come from non-endemic highlands to the cotton, sesame, and sorghum fields of Humara and Metema, the VL endemic lowlands on the Sudanese borderlands, during the harvesting season [17, 18]. So the prevalence of VL is dynamic as its mode of transmission changes according to the environment, socio-economic status, and immune status of the population [19]. HIV prevalence in Ethiopia has decreased from 1.5% to 2011 to 1.1% in 2015. As a result, despite declining HIV prevalence in the overall population, HIV prevalence among VL patients has remained disproportionately high. Various studies in Ethiopia have found that the prevalence of HIV-VL coinfection ranges from 18.1 to 48.5% [17, 18].

HIV prevalence in Ethiopia has decreased from 5.6% to 2005 to 2.6% in 2011 [20], with an estimated prevalence of 1.5% among adults in 2011 [21]. Even though HIV prevalence has decreased in the overall population, HIV prevalence among VL patients has remained disproportionately high [20]. VL accelerates HIV replication and disease progression, mainly by chronic immune stimulation [17, 18].

HIV prevalence rates of 20–40% among VL patients, the northwest districts of Ethiopia along the Sudanese border have the highest incidence of HIV and VL coinfection rates [22–24]. It is now a major problem in low-resource settings, where access to ART is limited. The highest burden worldwide is reported in northwest Ethiopia, where an estimated 20% of VL patients are HIV-co-infected. Young, male, migrant workers from the highlands that travel for seasonal work in this VL endemic area are the most at risk [25]. The study aimed to investigate the determinant risk factors of visceral leishmaniasis with HIV co-infected patients during the treatment period admitted at Metema Hospital using survival analysis.

## Methods

### Data source and study design

The secondary data was collected from the medical chart of visceral leishmaniasis with HIV co-infected patients from January 2015 to January 2021 at Metema Hospital using the ethical clearance approval letter obtained from the University of Gondar, College of Natural and Computational Science ethical approval committee (reference number: CNCS /10/15/4/2021) and a cross-sectional study design was conducted.

### Setting

The research was conducted at Metema Hospital. Metema was a town in north western Ethiopia, on the border with Sudan. This town was located in the North West Gondar Administrative Zone, Amara region, $897\ \text{km}$ North of Addis Ababa and $197\ \text{km}$ from the ancient city of Gondar.

### Study population

#### Inclusion criteria

The studies included only visceral leishmaniasis with HIV co-infected patients until death/censor with visceral leishmaniasis with HIV co-infection or cure with visceral leishmaniasis.

#### Exclusion criteria

All patients with incomplete data, during treatment, were excluded.

#### Sample size

A total of 153 visceral leishmaniases with HIV co-infection patients fulfilling the inclusion criteria in Metema hospital was used starting from January 2015 to January 2021.

### Dependent variables

The response or outcome variable was the survival time of Visceral leishmaniasis with HIV co-infection patients measured in days. The outcome variable was coded as 0 for censored and 1 for death.

### Independent variables

The socio-demographic factors related to the response variables were Age of patients in year, Sex of patients, Place of residence, Occupation and A Clinical variables were Baseline Weight(kg), Haemoglobin level (g/dl), Spleen size (cm), Baseline CD4^+^ cell count (cells/µL), Initial VL treatment regimen, treatment of VL-HIV Co-infection, history of tuberculosis, **t**reatment of VL-HIV Co-infection, Comorbidity(opportunistic infections), Baseline Body mass index (kg/m^2^), Nutritional Status, Temperature (^0^ C) and Bleeding.

### Statistical analysis

#### Survival data analysis

Survival model fitting to make an inference by non-parametric Model, semi-parametric Cox proportional hazard survival models. All inferences were conducted at 5% significance level data entered by SPSS and analysed by using STATA version 14 and R4.2.1 statistical software packages. The outcome variable of time until an incident occurs was a concern of the statistical technique of survival analysis for data analysis [26].

#### Nonparametric models

We use non-parametric methods, such as the Life-Table, the Nelson-Aalen, or the popular Kaplan–Meier estimate, to estimate (𝑡) as well as h(t). Kaplan-Meier survival analysis was used as the nonparametric approach to event history analysis [27], and a long-rank test to compare the survival difference between two or more groups [28].

#### Semi-parametric cox proportional hazard model

Cox proportional hazards (PH) model was one of the mathematical models designed for the analysis of time until an event or time between events. It shows the hazard at time t of an individual given the covariates. The hazard at the time was a product of baseline hazard function h0(t) which was only a function of time and exponential to the linear sum of βixi which is a function of time-independent covariates [29, 30].

The Cox Proportional Hazard model is given by;

1 $$\text{h}\left(\text{t}, \textrm{X},{\upbeta }\right)={\text{h}}_{0}\left(\textrm{t}\right)\text{exp}\left({\sum }_{\text{i}=1}^{\textrm{n}}{{\upbeta }}_{\text{i}}{\textrm{X}}_{\text{i}}\right)$$

Where $\text{h}\left(\text{t}, \text{X},{\upbeta }\right)$ was the hazard function at a time for a subject with covariate values X_1,_ X_2,_ X_3_… X_n_ and the estimated coefficients of the covariates of β_1_,β_2_,… β_n_. h_0_(t) was the baseline hazard function, which was the hazard function for an individual for which all the variables included in the model are zero, X= (X_1,_ X_2,_ X_3_……………. X_n)_ was the value of the vectors of the explanatory/predictor variables for a particular individual, ${\upbeta }({\upbeta }1,{\upbeta }2,\dots {\upbeta }\text{p})$ is a vector of the estimated coefficients of explanatory/predictor variables.

The Cox PH model’s exponential portion ensures that the fitted model will always give a non-negative hazard and by definition, a hazard function is between zero and plus infinity i.e. $0\le \text{h}\left(\text{t},\text{X},{\upbeta }\right)\le {\infty }$, then the hazard ratio for the two groups is defined as:

2 $$\text{H}\text{R}=\frac{\text{h}(\text{t}/\text{x}=1)}{\text{h}(\text{t}/\text{x}=0)}={\text{e}}^{{\upbeta }}$$

When HR = 1, it implies that the individuals in the two categories are at the same risk of getting the event, when HR > 1, it implies that the individuals in the first category (X = 1) are at a high risk of getting the event and if HR < 1, the individuals in the second category (X = 0) are at a high risk of getting the event.

#### Parameter estimation in cox-PH model

The Cox model likelihood function was called a “partial” likelihood function rather than a complete likelihood function. The fitted proportional hazard regression model was interpreted based on the hazard function (${e}^{\ddot{\widehat{\beta }}}.$)$\widehat{\beta }$ was the maximum partial likelihood estimator of β. The (1-α) 100% confidence interval for the estimated parameter is given as $\widehat{{\upbeta }}{\pm \text{z}}_{\frac{{\upalpha }}{2}}\text{*}\;\text{S.e}\left(\widehat{{\upbeta }}\right)$.

#### Method of variable selection

Model building starts from a single covariate analysis as suggested by Collett [31], who recommended the approach of first doing a single covariate analysis to “screen” out potentially significant variables for consideration in the multi-covariate model to identify the importance of each predictor.

## Results

### Descriptive statistics

Of the total patients of Visceral leishmaniasis with HIV (HIV1) co-infection included in the study, 5(3.27%) of the patients were female and 148(96.73%) were male. Considering sex, the proportion of death for females in visceral leishmaniasis with HIV (HIV1) co-infection is 10.53% and the proportion of death for male patients is 89.47%. Seeing age groups included in the study total sample of patients was 43.14%, and 56.86% of patients were their life expectancy from age group < 30 and ≥30 years respectively and the proportion of death for these age group were 42.11% and 57.89% respectively. Seeing the treatment type given for VL patients included in the study total sample of patients was 32.03%, 24.84% and 43.14% of patients were take Sodium Stibogluconate at dose 20 mg/kg/day with upper maximum daily dose limit of 850 mg, Sodium Stibogluconate and Paromomycin together with dose 15 mg/kg/day and Liposomal Amphotericin B(AmBisome) with dose 20 mg/kg respectively and the proportion of death for these VL treatment groups were 36.84%,31.58% and 31.58% respectively. Seeing the treatment given to VL-HIV Co-infection patients included in the study total sample of patients were 68.63% and 31.37% of patients were take Liposomal Amphotericin B(AmBisome) with dose 20–25 mg/kg and miltefosine with dose 100 mg/day the proportion of death for these VL-HIV(HIV1)Co-infection treatment groups were 26.32% and 73.68% respectively. From the total sample of VL-HIV(HIV1) Co-infection patients the proportion of having tuberculosis and not having tuberculosis were 36.60% and 63.40% respectively and their proportion of death were 36.84% and 63.16% respectively the rest of categorical variables were interpreted in the same manner. The mean of bassline weight, haemoglobin level, spleen size, and temperature of patients included in the study were 53.13 kg, 12.41 g/dl, 11.73 cm, and 37.63 ^0^ C with a standard deviation of 4.28, 2.01, 2.43and 1.79 respectively as shown in Table 1.

**Table 1:** Descriptive summary of sociodemographic and clinical covariates (at Metema Hospital, North West Gondar, during 2015–2021)

Variables	Categories	Status		Frequency(%)
		Censored(%)	Death(%)	
Age	< 30	58(43.28)	8(42.11)	66(43.14)
	≥30	76(56.72 )	11(57.89)	87 (56.86)
Sex	Female	3(2.24)	2(10.53)	5(3.27)
	Male	131(97.76)	17(89.47)	148(96.73)
Place of residence	Urban	29(21.64)	3(15.79)	32(20.92)
	Rural	105(78.36)	16(84.21)	121(79.08)
Occupation	Farmer	50(37.31)	5(26.32)	55(35.95)
	Migrant worker	72(53.73)	12(63.16)	84(54.90)
	Other	12(8.96)	2(10.53)	14(9.15)
Nutritional Status	Normal	61(45.52)	3(15.79)	64(41.83 )
	MAM	43(32.09)	4(21.05)	47(30.72)
	SAM	30(22.39)	12(63.16)	42(27.45)
Baseline CD4 + cell count (cells/µL)	< 100	62(46.27)	16(84.21)	78(50.98)
	≥ 100	72(53.73)	3(15.79)	75(49.02)
Initial VL treatment Regimen	SSG	42(31.34)	7(36.84)	49(32.03)
	SSG + PM	32(23.88)	6(31.58)	38(24.84)
	AmBisome	60(44.78)	6(31.58) |	66(43.14)
Body mass index	Normal	51 (38.06)	2 (10.53)	53 (34.64)
	Underweight	75 (55.97)	16 (84.21)	91 (59.48)
	Overweight	8(5.97)	1(5.26)	9 (5.88)
Bleeding	No	66(49.25)	4(21.05)	70(45.75)
	Yes	68(50.75)	15(78.95)	83(54.25)
**Treatment of VL-HIV Co-infection**	AmBisome	100(74.63)	5(26.32)	105(68.63)
	Miltefosine	34(25.37)	14(73.68)	48((31.37)
**Tuberculosis**	Yes	49(36.57)	7(36.84)	56(36.60)
	No	85(63.43)	12(63.16)	97(63.40)
**Tuberculosis treatment status**	Not on anti-tubercular treatment	85(63.43)	1(5.26)	86(56.21)
	History of anti-tubercular treatment	22(16.42)	8(42.11)	30(19.61)
	Currently on anti-tubercular treatment	27(20.15)	10(52.63)	37(24.18)
**Comorbidity**	Malaria	53(39.55)	9(47.37)	62(40.52)
	Tuberculosis	49(36.57)	7(36.84)	56(36.60)
	Any disease	32(23.88)	3(15.79)	35(22.88)
**Continuous covariate**				
Variables	Mean	Standard deviation (sd)		
Bassline Weight (kg)	53.13	4.28		
Hemoglobin level (g/dl)	12.41	2.01		
Spleen size (cm)	11.73	2.43		
Temperature (^0^ C)	37.63	1.79		

The accounted death and censored patients in the study period were 19 (12.42%) and 134 (87.58%), respectively and the mean survival time of patients followed were 68.115 days as shown in Table 2.

**Table 2:** Survival status and mean survival time for VL-HIV co-infected patients at Metema hospital

Death	Censored	Total	Mean of survival time	Sd	95% CI	
					Lower	upper
19(12.42%)	134(87.58%)	153	68.115	5.457	57.419	78.811

The period from the commencement of treatment until death served as the response variable. The overall mean estimated survival time of patients under the study was 68.115(95% CI: 57.419–78.811) days with a standard deviation of 5.457 as shown in Table 2.

### Non-parametric survival analysis

**Fig. 1:**
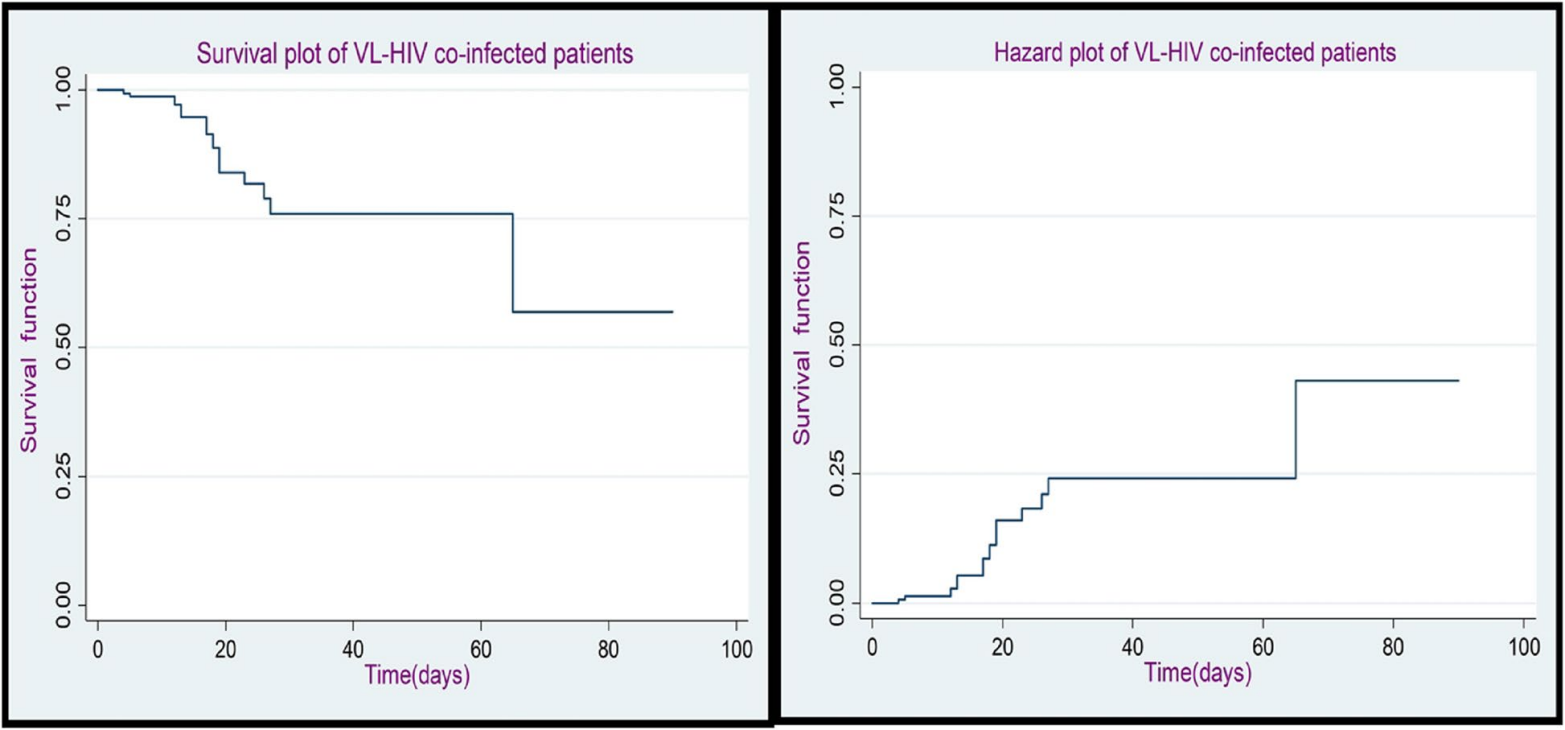
The overall K-M plot of survival and hazard function of VL-HIV co-infected patients at Metema Hospital, North West Gondar, during (2015–2021)

The graph of the estimate for the overall Kaplan-Meier survivor function represents that, relatively, a large number of the deaths occurred in the earlier days of treatment; and the same graph showed the decrement over a follow-up period as shown in (Fig. 1).

**Fig. 2:**
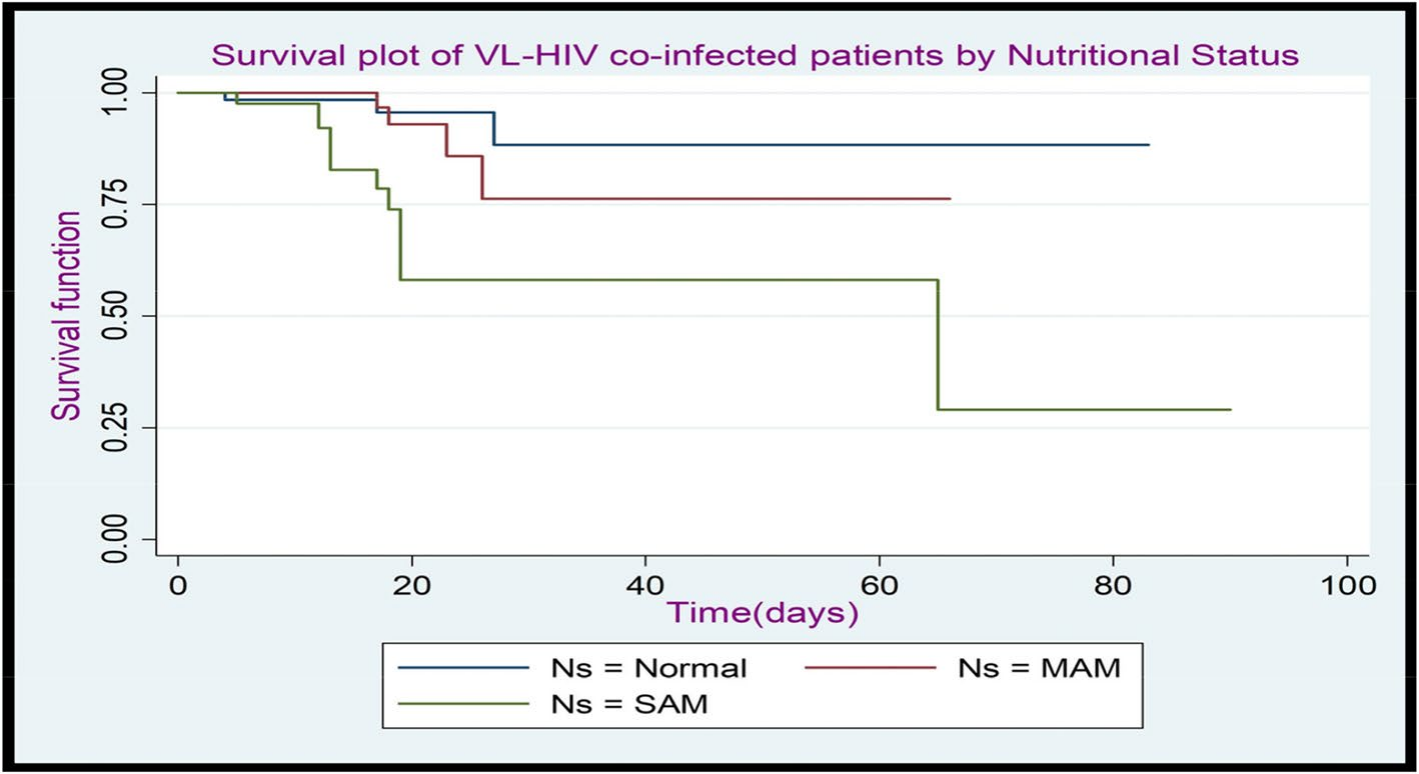
The K-M plot of the survival function of VL-HIV co-infected patients by nutritional status at Metema Hospital, North West Gondar, during (2015–2021)

Figure 2 Kaplan-Meier plot of the survival function shows that those patients with normal nutritional status were lies completely above moderate acute malnutrition, and moderate acute malnutrition lies above severe acute malnutrition patients. This suggests that those patients, with a nutritional status Normal, were better survival experience than moderate and severe acute malnutrition, and also moderate acute malnutrition had better survival experience than severe acute malnutrition patients.

**Fig. 3:**
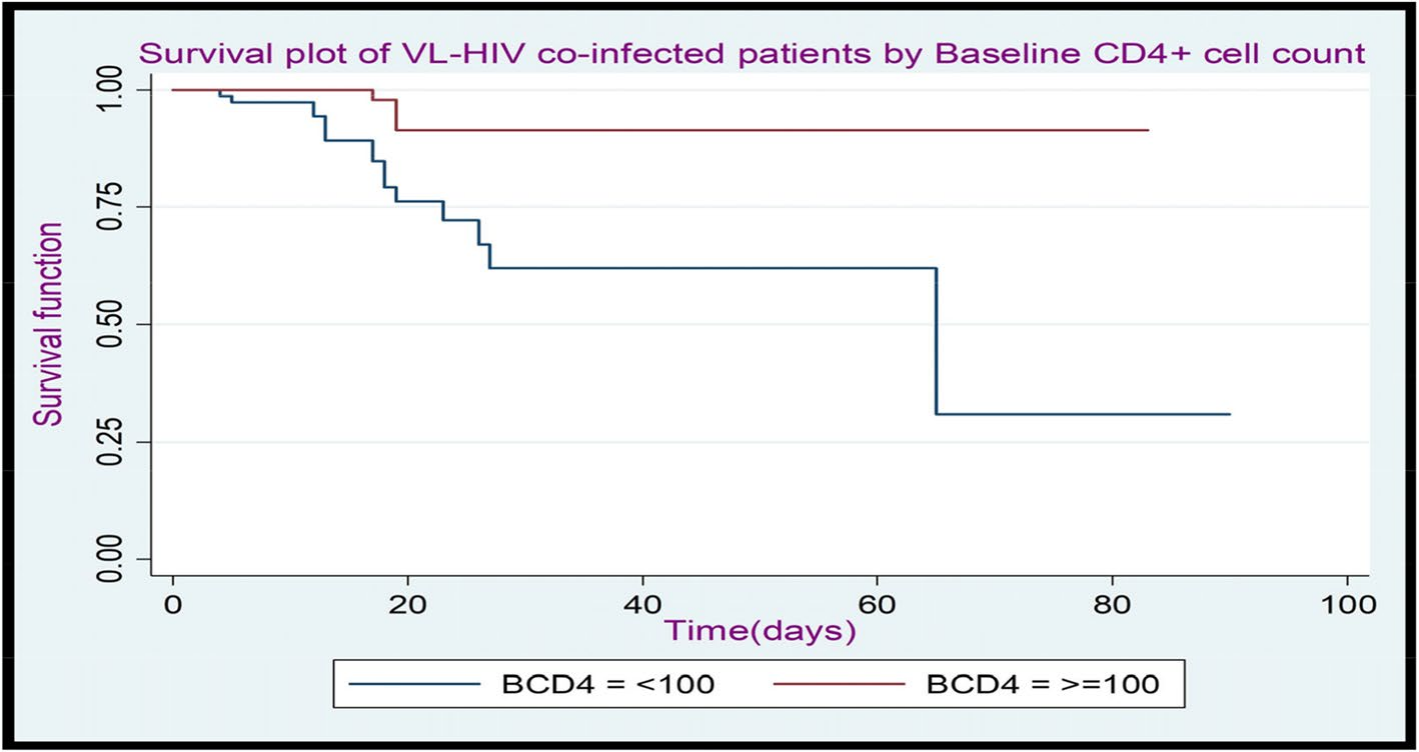
The K-M plot of the survival function of VL-HIV co-infected patients by Baseline CD4 + cell count at Metema Hospital, North West Gondar, during (2015–2021)

Figure 3 Kaplan-Meier plot of the survival function shows that in those patients their baseline CD4 + cell count ≥ 100 cells/µL lies completely above those patients < 100 cells/µL patients. This suggests that patients with a baseline CD4 + cell count ≥ 100 cells/µL would have a favourable survival experience than those with a baseline CD4 + cell count of < 100 cells/µ.

**Fig. 4:**
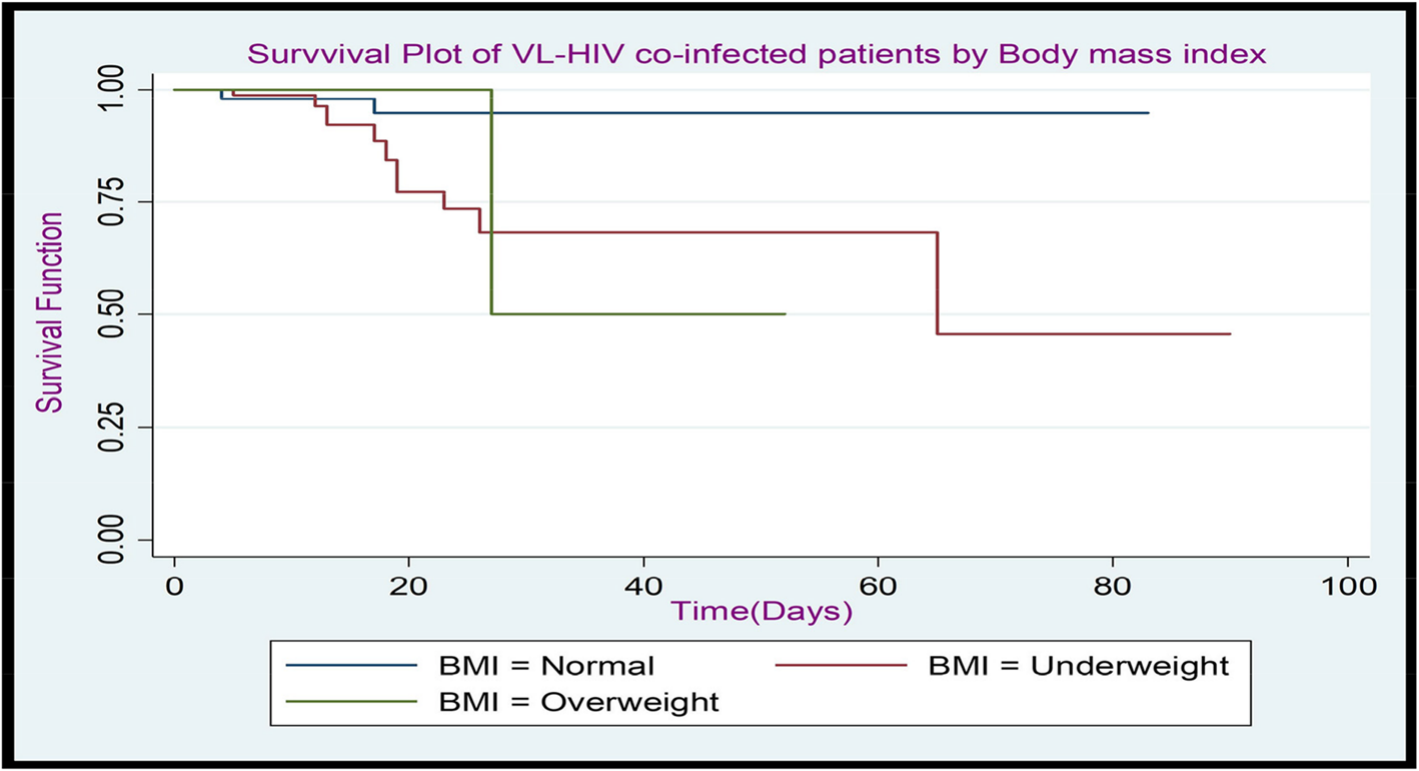
The K-M plot of the survival function of VL-HIV co-infected patients by body mass index at Metema Hospital, North West Gondar, during (2015–2021)

Figure 4 Kaplan-Meier plot of the survival function shows that those patients whose body mass index was normal were a better survival experience than underweight and overweight body mass index and additionally, individuals with low body mass indices have a higher rate of survival than those with high ones.

Comparing the differences among survival curves utilizing the graphical method was more or less subjective, so we employed a log-rank formal statistical test to check for significant differences among different categories of factors that had been demonstrated by using the Kaplan-Meier estimates of the survivor functions. The log-rank test statistical result survival time among different groups of predictors such as nutritional status, baseline CD4 + cell count and bleeding were significantly different in survival time and there was no significant difference in survival experience between the various categories of age group, sex, place of residence, occupation status, initial VL treatment regimen, treatment of VL-HIV Co-infection, history of tuberculosis, **t**reatment of VL-HIV Co-infection, Comorbidity(opportunistic infections), and body mass index of patients at 5% level of significance as shown above in Table 3.

**Table 3:** Results of the log-rank test for the categorical variables

Covariates	Chi-square	Df	(*P*-value) or sig.
Age	0.32	1	0.5699
Sex	1.78	1	0.1819
Place of residence	0.81	1	0.3673
Occupation	2.35	2	0.3091
Nutritional Status	14.94	2	0.0006^a^
Baseline CD4 + cell count	10.28	1	0.0013^a^
Initial VL treatment	1.75	2	0.4161
Body mass index	5.20	2	0.0743
Bleeding	5.58	1	0.0182^a^
Comorbidity	6.23	2	0.36
Tuberculosis	5.32	1	0.62
Tuberculosis treatment status	3.59	2	025
**Treatment of VL-HIV Co-infection**	2.01	1	0.49

^a^Indicate that the comparison difference was significant at a 5% significance level and df is the degree of freedom

### Uni-variable analysis of the Cox proportional hazards model

Univariate analyses were done using a 0.25 significance level. Covariates sex, occupation status, nutritional status, baseline CD4 + cell count, body mass index, bleeding, and the temperature was statistically significant at a 25% significance level in univariate analysis, so those variables are candidates for inclusion in the multivariable model.

### Multi-variable analysis of the Cox proportional hazards model

Nutritional status, baseline CD4 + cell count, and body mass index are significant at a 5% significance level in the final model and their parameter estimates of coefficients (β’s) for the covariates in the final model along with the associated standard error, hazard ratio, and 95% confidence interval for the hazard ratio is shown in Table 4.

**Table 4:** Final multi-variable Cox Proportional Hazard Modal for the VL-HIV co-infected patient (at Metema Hospital, North West Gondar, during 2015–2021)

Covariate	Category	Coef.(β)	Std. Err.(β)	*P*-Value	HR	95% CI for HR
Nutritional Status	Normal®				1	
	MAM	1.812005	1.153911	0.116	6.122708	(0.6378713 58.76978)
	SAM	3.23476	1.192169	0.007*	4.40027	(2.455061 262.7934)
Baseline CD4 + count	< 100 ®				1	
	≥ 100	-1.303932	0.6468098	0.044*	0.2714623	(0.0764089 0.9644395)
Body mass index	Normal ®				1	
	Underweight	1.542907	0.7517923	0.040*	4.678169	(1.970097 11.10872)
	Overweight	1.119183	1.225554	0.361	3.06235	(0.7477906 12.54093)
Obs	ll(null)	ll(model)	df	AIC	BIC	
153	-79.5905	-67.82027	5	145.6405	160.7927	

Std. *Err *Standard Error, *HR *Hazard Ratio, ® Reference group, *CI *Confidence Interval

* is significant at a 5% significance level

The final cox regression coefficients model was interpreted as looking at the effect of nutritional status after adjusting other confounding variables, the hazard of those patients whose nutritional status was severe acute malnutrition was 4.40027 times more likely than the hazard of those patients whose nutritional status was normal (HR = 4.40027, 95% CI = 2.455061–262.7934) indicating that the survival time was reduced by 40.027%. After adjusting other covariates, patients who had an underweight body mass index whose hazard rate was 4.678169 times more likely than that of normal-weight patients (HR = 4.678169, 95% CI **=** 1.970097–11.10872) indicating that the survival time was reduced by 67.8169%. Looking at baseline CD4 + cell count, after adjusting other covariates, patients who had Baseline CD4 + cell count **≥** 100 cells/µL were found to be associated with high survival time, whose hazard rate was 0.2237344 times less likely than that of those who had baseline CD4 + cell count **<** 100 cells/µL patients (HR **=** 0.2714623, 95% CI **=** 0.0764089 − 0.9644395) which mean the survival time of patients who had CD4 + cell count **≥** 100 cells/µL was increased by 72.85%.

## Discussion

In the study, 12.42% of the patients died even if they were taking treatment and using life table survival estimates and plot of overall Kaplan-Meier survivor function VL-HIV co-infection patients show a large number of the deaths occurred at the earlier days of treatment these imply that VL accelerates HIV replication and disease progression death this is in line with [17, 18].

Patients who had Baseline CD4 + Cell count greater than or equal to 100 cells/µL were found to be associated with high survival time, whose hazard rate was 0.2237344 times less likely than that of those who had Baseline CD4 + Cell count less than 100 cells/µL patients (HR = 0.2714623, 95% CI = 0.0764089 − 0.9644395) which means the survival time of patients who had CD4 + cell count greater than or equal to 100 cells/µL was increased by 72.85%. This indicates that those who have baseline CD4 + cell count of fewer than 100 cells/µL had lower survival time than those who have CD4 + cell count greater than or equal to 100 cells/µL patients. Our result is in line with other studies [32]. A Baseline CD4 + Cell count of fewer than 100 cells/µL (low CD4 + Cell count) was a significant predictor of death in HIV co-infected individuals with visceral leishmaniasis this is in line with [33] and Baseline CD4 + cell count of fewer than 100 cells/µL was linked to an increased probability of VL relapse. This finding was similar to those previously reported by [34].

Patients who had underweight body mass index were whose hazard rate was 4.678169 times more likely than that of normal-weight patients (HR = 4.678169, 95% CI = 1.970097–11.10872) indicating that the survival time was reduced by 67.8169%. So underweight body mass index was a significant factor in the mortality of visceral leishmaniasis with HIV co-infection patients these ideas were supported by [35]. Body mass index was a significant factor for mortality in Visceral leishmaniasis with HIV co-infection patients these finding was in line with the study conducted by [23, 36] and patients whose nutritional status severe acute malnutrition was 4.40027 times more likely than the hazard of those patients whose nutritional status was normal (HR = 4.40027,95% CI = 2.455061–262.7934) indicating that the survival time was reduced by 40.027%, so severe acute malnutrition was a significant factor for mortality Visceral leishmaniasis with HIV co-infection patients. Our result is in line with other studies [35, 37].

Bleeding tendency (Hemorrhage), and age greater than 20 years have a significant effect on Visceral leishmaniasis with HIV co-infection of patient mortality this result is in line with studies conducted by [23], Hemorrhage (bleeding tendency) was also the predictors of death conducted by [36] and Age, residence, and occupational (employment) status were independently associated with HIV-VL coinfection patients mortality in Northwest Ethiopia conducted by [17, 18].

In these Study, VL-HIV co-infection patients shows a large number of the deaths occurred at the earlier days of treatment these implies that VL accelerates HIV replication and disease progression death. So, this study is in line with a common immunopathological route between HIV and leishmaniasis promotes the growth of both infections and speeds up the progression of both VL and HIV [38] and these two infections can co-infect one another, which causes the disease to advance more quickly, become more severe, and have a worse therapeutic response. Numerous examples of HIV-Leishmania co-infection have been documented since the first visceral leishmaniasis (VL) and HIV case was published in 1999 from India [39] and it has been demonstrated that people with VL and HIV co-infection are very contagious [38].

## Conclusions

VL-HIV co-infection patients show a large number of deaths occurred in the earlier days of treatment these implies that VL accelerates HIV replication and disease progression death. The researcher suggests that people be aware of the burden posed by those risk factors and knowledgeable about the disease. So, the researcher recommended that to health workers implement primary health care in those patients and careful consideration of a neglected parasitic disease. The researcher recommended a further extension of these ideas to study time to death and its determinant factors of visceral leishmaniasis with HIV co-infected patients using Bayesian survival analysis.

## Data Availability

The corresponding author will provide the datasets used and analysed during the current research upon reasonable request.

## Abbreviations

AIDS: Acquired Immune Deficiency Syndrome
CD4: Cluster Differentiation Type 4
Cells/µL: Cells per Microliter
CI: Confidence Interval
DF: Degree of Freedom
g/dl: Gram per decilitre
HIV: Human immunodeficiency virus
HR: Hazard Ratio
K-M: Kaplan–Meier
MAM: Moderate Acute Malnutrition
PH: Proportional Hazard
PM: Paromomycin
SAM: Severe Acute Malnutrition
SD: Standard Deviation
SPSS: Statistical Packages for Social Science
SSG: Sodium Stibogluconate
VL: Visceral Leishmaniasis
WHO: World Health Organization
